# Cognitive Impairment in Multiple Sclerosis

**DOI:** 10.3390/bioengineering10070871

**Published:** 2023-07-23

**Authors:** Kenneth Maiese

**Affiliations:** Cellular and Molecular Signaling, New York, NY 10022, USA; wntin75@yahoo.com

**Keywords:** APOE-ε4, apoptosis, autophagy, COVID-19, dementia, FoxO, mTOR, multiple sclerosis, nicotinamide, SIRT1

## Abstract

Almost three million individuals suffer from multiple sclerosis (MS) throughout the world, a demyelinating disease in the nervous system with increased prevalence over the last five decades, and is now being recognized as one significant etiology of cognitive loss and dementia. Presently, disease modifying therapies can limit the rate of relapse and potentially reduce brain volume loss in patients with MS, but unfortunately cannot prevent disease progression or the onset of cognitive disability. Innovative strategies are therefore required to address areas of inflammation, immune cell activation, and cell survival that involve novel pathways of programmed cell death, mammalian forkhead transcription factors (FoxOs), the mechanistic target of rapamycin (mTOR), AMP activated protein kinase (AMPK), the silent mating type information regulation 2 homolog 1 (*Saccharomyces cerevisiae*) (SIRT1), and associated pathways with the apolipoprotein E (APOE-ε4) gene and severe acute respiratory syndrome coronavirus (SARS-CoV-2). These pathways are intertwined at multiple levels and can involve metabolic oversight with cellular metabolism dependent upon nicotinamide adenine dinucleotide (NAD+). Insight into the mechanisms of these pathways can provide new avenues of discovery for the therapeutic treatment of dementia and loss in cognition that occurs during MS.

## 1. Increased Lifespan, Aging, Cellular Senescence, and Nervous System Disorders

Life expectancy is increasing throughout the globe, and individual lifespan is expected to reach 80 years of age [1,2,3,4,5,6]. There also is a one percent decrease in the age-adjusted death rate from the years 2000 through 2011 [7], and the number of individuals over the age of 65 has doubled during the last 50 years [8]. In large developing countries, such as China and India, the elderly population is expected to increase from five to ten percent in the coming years [9,10]. The observed increase in lifespan for individuals has several components that have resulted from measures focused on improving sanitation and reducing infection, allowing greater access to higher quality healthcare, the incorporation of early diagnostic and preventive measures, and quickly identifying individuals susceptible to both acute and chronic illnesses [5,11,12,13,14,15,16,17,18,19,20,21,22].

With the rise in longevity and lifespan, non-communicable diseases (NCDs) have increased in prevalence as well [2,5,23,24,25,26]. NCDs lead to seventy to eighty percent of annual deaths; that equals greater than forty million individuals dying each year [27,28]. Of the individuals affected by NCDs, fifteen million people at minimum are in the age range between thirty and sixty-nine years of age. With NCDs, there exists a disproportionate impact on lower-income countries [2,23,24,25]. In among ten to fifteen percent of people in high-net-worth nations, NCDs involve people that are below sixty years of age, but in low- and middle-net-worth nations, NCDs impact a larger segment of individuals that involves at least thirty-three percent of the population who are below sixty.

Nervous system diseases comprise a large portion of NCDs [2,29,30,31,32,33,34,35,36,37,38,39,40]. Neurodegenerative disorders impact more than fifteen percent of the global population and comprise greater than six hundred disease entities that can result in severe disability and death [10,37,41,42,43,44,45,46,47]. More than seven million individuals die each year from diseases of the nervous system [2,18]. A significant factor that plays a role in the onset and progression of neurodegenerative disorders is the cellular mechanisms of aging [21,40,48,49,50,51,52,53,54]. At the cellular level, telomeres (TLs), which are complexes of deoxyribonucleic acid (DNA), can affect aging, the onset of cell senescence, and the development of neurodegenerative disorders [55,56,57]. TLs reside at the end of chromosomes and can modulate cell replication, cellular lifespan, and protection for the DNA of the genome. TLs have greater than 2000 repetitions of non-coding double-stranded DNA with the sequence “TTAGGG”, and are completed with a guanine rich single-stranded DNA [55,58]. Several protein complexes are associated with TLs, which include telosome, shelterin, and CTC1-STN1-TEN1 (CST). These complexes are necessary to regulate the activity of TLs and provide stability for TLs. During cellular division, the telomerase protein can become active to maintain TL length through the addition of tandem repeat ribonucleic acid (RNA) templates, since a portion of the TLs length is lost in the amount of approximately 25–200 base pairs [59,60,61,62]. If telomerase function in somatic cells becomes no longer viable or the TLs become excessively short with less than 500 base pairs, cell proliferation is blocked and cell senescence results [6,63,64,65,66,67,68,69,70,71,72,73,74,75,76]. If cell senescence does ensue, systems of the body cannot repair themselves, which leads to advancement of aging processes. As a result, an inability to remove cells that are senescent by the immune system also may stimulate tumorigenesis [6,40,63,64,67,68,72,75,77]. The release of reactive oxygen species (ROS) during oxidative stress can occur during the shortening of TLs and the onset of cell senescence. Oxidative stress exposure ultimately affects cell survival and mitochondrial organelle function through TL shortening and the onset of cell senescence [21,52,59,68,72,74,75,77,78,79,80,81,82,83,84,85,86,87,88,89,90,91,92,93,94].

These processes related to ROS, mitochondrial injury, the shortening of TLs, and cell senescence can hasten aging, and also promote the onset of neurodegenerative disorders such as dementia and Alzheimer’s disease (AD) [55,58,67,69,71,73,95]. Individuals with shorter leukocyte TL lengths can have an increased risk of dementia and AD [56]. Leukocyte TL length has been tied to observations on cerebral magnetic resonance imaging (MRI). Longer TL lengths may be protective against dementia and nervous system injury due to increased hippocampal grey matter volumes, lower volumes of white matter hyperintensities, and lower presences of basal ganglia iron on MRI under these circumstances [57].

## 2. Cognitive Impairment in Neurodegeneration

All populations in all nations are affected by dementia, and it is the 7th leading cause of death per the World Health Organization [28]. Dementia is considered to be significantly underdiagnosed, with delays in recognition of cognitive loss in individuals. Evaluation and treatment of impaired cognition may not start until one or two years later following the performance of a correct investigation [96,97]. If one considers disorders such as AD, familial cases of AD (FAD) affect a much smaller proportion of the population. FAD is an autosomal dominant form of a mutated amyloid precursor protein (APP) gene, and occurs in approximately 200 families worldwide [71,95,98,99,100,101,102]. FAD occurs most often prior to age 55, and results from variable single-gene mutations on chromosomes 1, 14, and 21 [23,103,104]. In contrast, more than ten percent of the global population over the age of 65 is affected with the sporadic form of AD; the ε4 allele of the apolipoprotein E (APOE) gene results in heightened risk, and at least 60 percent of individuals with dementia have AD [73,98,105,106,107,108,109]. In the United States (US) alone, greater than 6 million individuals have AD, and an additional 4 million individuals are under treatment at an annual cost of 4 billion United States dollars (USD) [86,97,110,111,112]. As age and lifespan increases in the population, it is expected that the number of individuals with AD will increase to 30 million individuals over the next 15 to 20 years [69,105,113]. At present, fifty million people in the world, or five percent of the global population, have dementia [24,31,73,95,98,106,108,114]. When one considers the year 2030, 82 million people will endure disability from cognitive loss. In the year 2050, more than 155 million individuals will suffer from dementia [10,22,23,24].

In regard to the cost to care for cognitive impairment, dementia care has a cost factor of greater than $800 billion USD a year [28]. Social services and medical needs in the year 2030 will require 2 trillion USD every year in the US alone, as currently greater than 4 million individuals require resources of 3.8 billion USD total every year for care. Although possibly underestimated, it is predicted that market needs for AD will exceed 11 billion USD [18,105,115]. Additional expenses also come under consideration, such as the need to employ close to 70 million social care individuals and health care staff for the essential need of companion and adult behavior care, as well as outreach systems to address social needs [27,28,116]. Furthermore, in addition to AD, other neurodegenerative disorders may also have a complicated presentation with cognitive loss. Parkinson’s disease (PD) is ranked as a second nervous system disease that leads to dementia when compared to AD. PD is a movement disorder that leads to resting tremors, rigidity, and bradykinesia, and is characterized by the loss of dopaminergic neurons in the substantia nigra [22,29,69,111,117,118,119,120,121,122]. More than 10 million individuals suffer from PD in the world, which includes 50,000 new cases annually in the US, and many suffer from cognitive loss [117,123]. The number of individuals with PD is expected to double by the year 2030 [10,18,124,125]. More than $52 billion USD are spent in the US alone per year, with an annual cost per patient that approaches approximately $25,000 US dollars per year.

## 3. Cognitive Loss and Dementia That Can Occur in Multiple Sclerosis

**Figure 1:**
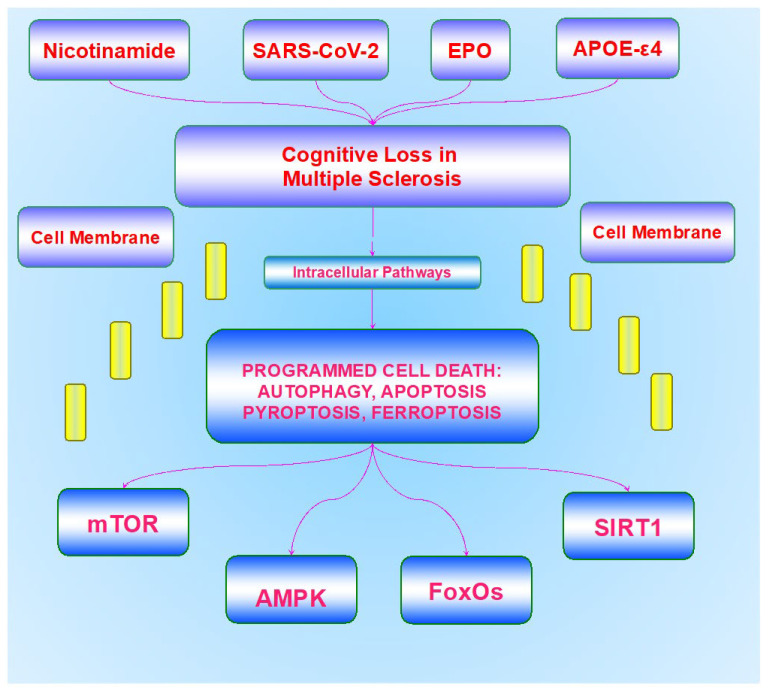
Innovative pathways for the treatment of cognitive loss in multiple sclerosis. A number of new strategies can address multiple areas of cognitive loss and disability for multiple sclerosis that involve intracellular pathways inside the cell membrane (yellow bars), consisting of programmed cell death with autophagy, apoptosis, pyroptosis, and ferroptosis, mammalian forkhead transcription factors (FoxOs), the mechanistic target of rapamycin (mTOR), AMP activated protein kinase (AMPK), and the silent mating type information regulation 2 homolog 1 (*Saccharomyces cerevisiae*) (SIRT1). External extracellular modulators outside of the cell membrane involve nicotinamide adenine dinucleotide (NAD+) through nicotinamide, trophic factor exposure such as with erythropoietin (EPO), and associated risk factor exposure with the apolipoprotein E-ε4 (APOE-ε4) gene and severe acute respiratory syndrome coronavirus (SARS-CoV-2).

Interestingly, individuals with the neurodegenerative disorder multiple sclerosis (MS) also are significantly impacted by cognitive loss and dementia (Figure 1). Demyelinating disorders, such as MS, affect a significant proportion of the world’s population [38,126,127,128,129,130,131,132,133,134,135] (Table 1). MS is considered to be the most common demyelinating disorder that affects the immune system and the central nervous system through the function of myelin-producing cells [117,127,134,136,137,138,139,140]. At least 2.5 million individuals suffer from MS around the world, and a continual increase in the prevalence of MS has occurred over the prior five decades. Women are affected more than men by MS [133].

While cognitive loss has not been previously considered as a primary disability in the MS patient group, it is increasingly becoming recognized. Early descriptions by Charcot noted that patients with MS were slow in formulation of their thoughts and had difficulty retrieving their memories [141]. Observations of cognitive loss have become more common and refined over the years. In some patient cohorts of MS that included both men and women, the prominent clinical phenotype was characterized by severe cognitive impairment that was progressive in nature [142]. In other recent studies, cognitive impairment may not be so severe, but definitely present with word finding difficulty and visual object naming [143]. The reduction in formation processing speed that is observed with cognitive loss in MS can be independent of mood disorders, and impairments in visual object meaning perception may appear uniquely in individuals with MS [143]. It is estimated that loss of cognitive function in MS occurs in approximately sixty-five percent of individuals and can affect processing of information, attention, memory recall, and other executive functions [144]. With cognitive loss in MS, cortical atrophy loss and abnormal cortical integrity occurs with cognitive behavior therapy programs, exercise treatment, and education programs still at an infancy stage to treat memory loss in MS [144]. Treatments based on LINGO-1 (LRR and Ig domain containing NOGO receptor interacting protein) antagonism and protein kinase B (Akt) activation in experimental models of cognitive loss in MS may assist to maintain memory function through the promotion of oligodendrocyte differentiation and myelination [145]. Mood disorders that include depression, apathy, and anxiety can accompany the cognitive loss in MS similar to presentations seen in AD [99,146,147,148,149,150,151]. The cognitive impairment in MS patients also may play a significant role in preventing individuals from returning to prior levels of functioning and the workforce [152]. Virus exposure also may contribute to neurodegenerative disorders such as AD and MS. In the case of MS, Epstein-Barr virus has an increased risk of leading to MS and cognitive loss that may occur up to 15 years after initial infection [43].

Risk factors for this cognitive loss in MS may share common ground with those of AD (Figure 1). For example, heightened risk of late-onset AD is present in conjunction with the ε4 allele of the apolipoprotein E (APOE-ε4) gene [105,153,154,155]. If someone has two ε4 alleles, they could potentially have a 20 times greater risk of suffering from AD compared to individuals without two ε4 alleles. The isoform APOE-ε4 cannot destroy β-amyloid (Aβ) in the brain, which may lead to an increased risk for the development of AD [153,154,156,157,158,159]. However, APOE in the body is important for cellular function. APOE originates in hepatic cells, is necessary for transporting cholesterol, triglycerides, and phospholipids in the body, as well as for regulating lipid homeostasis [160,161]. APOE in the central nervous system begins its production in astrocytes and facilitates the transfer of cholesterol to neuronal cells that are dependent upon APOE receptors [105,156]. APOE, at times, can destroy cerebral Aβ through apoptotic cellular pathways. Phosphatidylserine (PS) membrane exposure [162,163], an initial phase in apoptotic cell death, may be related to Aβ aggregation. Isoforms of APOE that are exclusive of APOE-ε4 have been suggested to block Aβ aggregation through the exposure of PS membranes [158]. These observations may be relevant for MS and cognitive loss in this disorder. APOE-ε4 may be associated with the onset and progression of cognitive impairment in patients with MS. Individuals with MS and APOE-ε4 experienced delayed rates to stimuli and difficulties with cognitive function [164]. In patients with optic neuritis [165,166], a common occurrence in almost half of patents with MS. APOE serum levels were significantly higher than in control patient groups and the *APOE* ε3/ε3 genotype may increase the risk of developing optic neuritis in males [167]. In addition to APOE-ε4, MS may share other cellular pathways with AD that lead to cognitive loss such as Aβ [37,102,168,169,170,171,172]. In studies that examine cerebrospinal fluid biomarkers, changes in Aβ_42_ as seen in AD can also predict early cognitive decline in MS [173]. Furthermore, the presence of tau seeding, which can lead to AD pathology [37,98,100,102,105,174,175,176], has been observed in the brains of patients with MS [177], and tau also may contribute to impaired oligodendrocyte maturation and pathological changes that may foster demyelination [178].

## 4. Innovative Therapeutic Strategies for Cognitive Loss in Multiple Sclerosis

MS can lead to multiple disabilities for individuals with the onset of cognitive impairment, behavioral difficulties, blindness, loss of motor function, sensory dysfunction, and loss of coordination. Given the spectrum of the presentations for MS, it should be no surprise that a vast array of cellular mechanisms may foster the onset and progression of MS. Pathways that involve inflammatory mediators, demyelination and remyelination pathways, oxidative stress, blood-brain barrier impairment, viral antigens, and cellular metabolism, which are dependent upon nicotinamide adenine dinucleotide (NAD+), have been tied to the underlying pathology of MS [10,29,31,33,39,43,72,77,85,86,99,100,115,162,171,179,180,181,182,183,184,185,186,187,188,189,190,191,192,193,194,195,196,197,198]. Although new treatments that address MS, known as disease modifying therapies (DMTs), can limit the rate of relapse in patients with relapsing–remitting MS, DMTs cannot prevent disease progression. In addition, brain volume loss may be another component of dementia and further disability that may be independent of disease activity. Early initiation of DMTs may slow the progression of brain volume loss, but cognitive disability may continue to ensue [199]. In light of these considerations to address both disease onset and progression in MS, novel pathways of discovery are required (Table 1). Innovative avenues that can address underlying cellular disease mechanisms may provide fruitful options for future clinical care that involve programmed cell death pathways, mammalian forkhead transcription factors (FoxOs), the mechanistic target of rapamycin (mTOR), AMP activated protein kinase (AMPK), the silent mating type information regulation 2 homolog 1 (*Saccharomyces cerevisiae*) (SIRT1), and associated pathways with the apolipoprotein E (APOE-ε4) gene and severe acute respiratory syndrome coronavirus (SARS-CoV-2) (Figure 1).

## 5. Autophagy, Apoptosis, Pyroptosis, and Ferroptosis Involvement in Multiple Sclerosis

Recent studies have identified a significant role for pathways of programmed cell death that can ultimately control cell survival and cognitive impairment during MS [46,61,128,130,132,134,200,201,202] (Table 1). Programmed cell death pathways that involve autophagy, apoptosis, pyroptosis, and ferroptosis can influence cell survival during inflammation [34,39,66,73,74,85,87,203,204,205,206,207,208,209,210,211,212,213], oxidative stress [10,45,77,86,89,93,119,185,214,215,216,217,218,219], ischemia [219,220,221,222,223], and mitochondrial dysfunction [32,83,85,115,198,224,225,226,227,228,229,230,231,232] (Figure 1). Disorders of cellular metabolism that can lead to cognitive loss and other impairments, such as diabetes mellitus (DM), are also intimately tied to pathways of programmed cell death [24,72,115,208,214,216,219,224,233,234,235,236,237,238,239,240,241].

In general, autophagy consists of the recycling of cytoplasmic organelles and proteins that can lead to the remodeling and formation of new tissue structures [34,44,61,100,169,202,242,243,244,245]. Although the process of macroautophagy is usually described, other subsets of autophagy exist that include microautophagy and chaperone-mediated autophagy. Macroautophagy refers to the recycling of organelles by forming autophagosomes consisting of cytoplasmic proteins and organelles that can be combined with lysosomes for eventual degradation, which are then used during recycling processes [71,73,97,107,197,246]. Microautophagy employs the invagination of lysosomal membranes to sequester and consume components of the cell cytoplasm [105,229]. In relation to chaperone-mediated autophagy, this form of autophagy utilizes cytosolic chaperones that oversee the transportation of cytoplasmic structures across lysosomal membranes [10,124,198,246,247,248,249].

During MS, autophagy may have an important role in the regulation of oligodendrocyte development, myelination, and activation of microglia [130,134,201]. Autophagy in demyelinating diseases also has a vital role during oxidative stress and ROS generation [198,202,250,251,252,253,254]. The induction of ROS can result in alterations in mitochondrial function [2,79,85,87,255] that may also impact cognitive loss. In addition, during infections, the autophagy-lysosome pathway can increase inflammatory reactions. An example of this is the severe acute respiratory syndrome coronavirus (SARS-CoV-2) [85,110,115,169,239,251,256,257]. Recent work has shown that exposure to infectious agents, such as SARS-CoV-2 as part of coronavirus disease 2019 (COVID-19), may lead to increased death rates in patients with MS [258].

With the activation of apoptosis, myelin injury, recovery, and cell death can be affected in MS. This may be mediated through the generation of ROS and oxidative stress that leads to mitochondrial dysfunction, demyelination, and neuronal axonal loss [21,53,87,198,200,232,241,259]. Apoptosis is a cell death pathway that consists of an early phase and a later phase [22,45,61,202,260]. During the early phase, phosphatidylserine (PS) membrane asymmetry loss occurs on the plasma membrane [261,262,263,264,265]. With the loss of membrane PS asymmetry during cell injury, inflammatory cells that reside in the nervous system, such as microglia, are attracted to injured cells and can remove them from the nervous system, resulting in nervous system dysfunction [261,266,267,268,269]. However, the loss of membrane PS asymmetry is reversible, and if membrane asymmetry is restored, injured cells are then given the ability to recover, and not be engulfed by inflammatory cells [197,270,271,272]. With the later phase that involves deoxyribonucleic acid (DNA) degradation in the cell [34,40,77,226,273,274,275], caspase activation plays a prominent role [15,77,194,206,210,273,276,277,278]. This process of caspase activation and DNA destruction is irreversible [259]. Limiting apoptotic cell death may minimize memory loss and cognition during both acute and chronic insults [34,93,96,169,172,185,276,279]. This may occur during measures that reduce inflammatory pathways and affect neuronal and oligodendrocyte survival [9,108,132,187,252,276,279,280,281,282], as well as cognitive performance [25,283,284,285].

Pyroptosis also may have an important function in MS and the treatment of patients. Increased inflammasome expression has been associated with potential treatment failures in MS patients [286], as well as the increased inflammatory activity of cytokine release and immune cell activity in experimental models [134]. Pyroptosis is a programmed cell death pathway that can oversee inflammatory cell activation in the nervous system [34,66,169,212,213]. Pyroptosis is initiated with the production of a supramolecular complex, known as the pyroptosome or the inflammasome. It should be noted that inflammasomes are cytosolic oligomers of multiple proteins, and the inflammasome family with NLRs (nucleotide-binding oligomerization domain and leucine-rich repeat-containing receptors) contains NLRP1, NLRP3, NLRP6, and NLRC4. Inflammasome activation can occur through pattern recognition receptors responding to the damage-associated molecular pattern (DAMP) of host cells or pathogen-associated molecular pattern (PAMP) in the microbial family [34,66,134,185,212,213,286,287,288,289,290]. In pyroptosis, the inflammasome is responsible for caspase 5, caspase 4, and caspase 1 activation. Pyroptosis also results in plasma membrane permeabilization involving protein family members of gasdermin. Gasdermin proteins consist of an *N*-terminal domain with intrinsic pore-forming properties and a *C*-terminal domain that inhibit *N*-terminal domain pore formation. Plasma pores result from fragmentation of the *N*-terminal domain during breakage of the association of the *N*-terminal and the *C*-terminal domains. At this point, interleukin-1 family members and other cytokines are released through cell membranes that can either assist or obstruct cell survival, which requires gasdermin. Gasdermin proteins are necessary to generate membrane pores since family members of interleukin-1 family members are absent of peptides in the membrane to generate membrane pores [24,34,220]. Once the cell membranes are open, DAMP entities involving DNA and adenosine triphosphate (ATP) are released. In the canonical inflammasome path, DAMPs activate the NLR family pyrin domain containing 3 (NLRP3) inflammasome. In contrast, caspase 4 and caspase 5 are activated, such as in Gram-negative bacterial infections, with noncanonical inflammasomes involving lipopolysaccharide proteins. Pyroptosis in conjunction with necroptosis and apoptosis can result in inflammation that generates elevated cytokine release and cell injury [212,291]. In the setting of ROS release and oxidative stress, pyroptosis can generate a severe inflammatory response that affects both neuronal and vascular cells, impairing cognition [5,85,86,94,106,185,292].

Ferroptosis, also an important mediator of the programmed cell death pathway, has been recently linked to MS and cognitive loss [169,293,294]. Ferroptosis results in intracellular iron accumulation and loss of glutathione homeostasis [295]. With the failure of glutathione-dependent oxidative stress defenses, ferroptosis leads to excessive lipid peroxidation and subsequent cell death. Under some conditions, it is believed that ferroptosis during MS results in the development of pathogenic T lymphocytes that impair the function of both neuronal and glial cells [296]. Ferroptosis is also involved in tumorigenesis [297] and cardiomyocyte injury as well [298].

## 6. Mammalian Forkhead Transcription Factors and Multiple Sclerosis

Given that mammalian forkhead transcription factors (FoxOs) can have an important relationship to cell death pathways during neurodegenerative disorders [2,5,49,259,260,299,300,301,302,303,304], they are increasingly being recognized as potential therapeutic targets for MS (Figure 1). In particular, the mammalian FOXO proteins of the O class can lead to neuronal cell death through apoptosis and autophagy activation [5,49,50,68,128,203,250,301,305,306,307,308,309,310,311,312,313,314,315]. Other studies suggest that the progressive course of MS may be associated with epigenetic changes of DNA methylation that are dependent upon genetic variations of FOXOs, such as FoxO1 and FoxO3a [128] (Table 1).

FoxOs are especially interesting since they can influence behavior and memory loss [18,203,309,316]. More than one hundred genes in the forkhead family and nineteen human subgroups have been described. They consist of *FOXA* to *FOXS* following the discovery of the *Drosophila melanogaster gene forkhead*. Forkhead proteins are also termed forkhead in rhabdomyosarcoma (FKHR) (FOXO1), FKHRL1 (forkhead in rhabdomyosarcoma like protein 1) (FOXO3a), the *Drosophila* gene fork head (*fkh*), Forkhead Related Activator (FREAC)-1 and -2, and the acute leukemia fusion gene located in chromosome X (*AFX*) (*FOXO4*) [231,259]. Numbers of Arabic origin are employed for the nomenclature with “Fox”, then a subclass or subgroup letter is listed, and then the member number is presented within the subclass [186,259]. Letters are capitalized for human Fox proteins. Only the initial letter is listed as uppercase for the mouse; for all other chordates, the initial and subclass letters are in uppercase [259,317,318]. Given that FoxO proteins are transcription factors, they bind to deoxyribonucleic acid (DNA) through the FoxO-recognized element in the *C*-terminal basic region of the forkhead DNA binding domain. In the α-helix H3 recognition region, fourteen protein-DNA contacts modulate the gene expression of targets [319]. A number of factors control DNA and forkhead interactions that involve FoxO protein phosphorylation or acetylation, FoxO protein compartmentalization in the nucleus, and alteration of electrostatic changes [259,277,306,307,320,321].

FoxO proteins are expressed throughout the body. In the nervous system, mammalian FOXO proteins of the O class that consist of FOXO1, FOXO3, FOXO4, and FOXO6 can lead to nervous system disorders [5,49,50,317,322,323]. FoxO proteins are also linked to metabolic function that can affect neurodegenerative disorders [17,24,169]. The function of FoxO proteins is conserved among multiple species, which include *Caenorhabditis elegans, Drosophila melanogaster*, and mammals. FoxO proteins are homologous to the transcription factor Dauer Formation-16 (DAF-16) in the worm *Caenorhabditis elegans*, affecting metabolic insulin signaling, cell cycle regulation, cell survival, and may also oversee lifespan extension [324,325,326]. FoxO proteins appear to have a selective expression in the nervous system, which may offer insight into the biology for specific FoxO proteins. For example, FoxO3 may affect auditory synaptic transmission [327], cerebral endothelial vascular cell survival [269,328], cerebral traumatic injury [316], cell survival during oxidative stress [53,329], and hippocampal degeneration [330,331]. FoxO6 modulates gluconeogenesis [332] and memory consolidation [322], and is present in several regions of the brain, such as the hippocampus, the amygdala, and the nucleus accumbens [333,334]. FoxO1 can have a more diverse role in gastric cancer [335], glaucoma [313], renal disease [315], astrocyte survival [336], motor and memory pathways in the striatum and sub-regions of the hippocampus [333], ischemic brain injury [337], and attenuation of Aβ accumulation and tau phosphorylation in the brain [338].

The structure of FoxOs is interesting. The forkhead box (FOX) family of genes have a butterfly-like appearance on X-ray crystallography and nuclear magnetic resonance imaging with a conserved forkhead domain (the “forkhead box”), described as a “winged helix”. Three α-helices, three β-sheets, and two loops make up the “winged helix” that appears to be unique for the forkhead family, since other winged helix domains do not fall under the Fox protein family.

FoxOs are modulated by epigenetic and post-translation protein modifications that involve phosphorylation [232,259,339], ubiquitylation [331], and acetylation [306,320,321]. Phosphorylation of FoxOs is controlled by Akt [72,340] to prevent translation to the nucleus through association with 14-3-3 proteins, inhibit gene transcription, and block apoptosis [259,312,330]. Once FoxO proteins such as FoxO3a are activated, cytochrome c release can occur with caspase-induced apoptotic death [262,341,342,343]. Akt also has a secondary regulatory mechanism that controls FoxO proteins to prevent caspase activity. Although FoxO3a is phosphorylated in the presence of oxidative stress, cleavage of FoxO3a does not occur during Akt inhibition of caspase 3 activity, preventing apoptosis [344] and the generation of “pro-apoptotic” amino-terminal (Nt) fragments following FoxO3a cleavage [345]. Akt also results in the ubiquitination and degradation of FoxOs through the 26S proteasome. In regard to acetylation, FoxOs are acetylated by histone acetyltransferases that include the CREB-binding protein (CBP), the CBP-associated factor, and p300. Once FoxOs undergo acetylation, FoxO proteins are able to transfer to the cell nucleus, but FoxO protein activity and DNA binding is somewhat inhibited by acetylation of lysine residues on FoxO proteins [306,320,321,346], and acetylation of FoxOs also leads to phosphorylation of FoxOs by Akt [347,348].

FoxOs are intimately tied to the pathways of programmed cell death. Blockade of FoxO transcription factor activity can inhibit microglial cell apoptotic death during ROS and Aβ exposure [343,344,349,350,351], foster the protective effects of metabotropic glutamate receptors [259,352], and prevent neuronal apoptotic cell loss through nicotinamide adenine dinucleotide (NAD^+^) precursors [86,162,198,213,353,354,355,356,357,358]. For example, nicotinamide can block FoxO protein activity [329,359] and is protective through two mechanisms of post-translational modification of FoxO3a [50,186,317]. Nicotinamide can not only maintain phosphorylation of FoxO3a and inhibit its activity to potentially block caspase 3 activity [329], but also it can reduce caspase activity and preserve the integrity of the FoxO3a protein to block FoxO3a proteolysis; that would normally lead to the generation of “pro-apoptotic” amino-terminal (Nt) fragments. Furthermore, growth factors such as erythropoietin (EPO) [119,169,187,340,360,361,362,363,364,365] are also dependent upon FoxOs to prevent apoptotic cell loss. Through post-translational changes, EPO phosphorylates FoxO3a [366] to sequester FoxO3a in the cell cytoplasm through the association with 14-3-3 protein [269,367]. EPO can also remove FOXO3a and FOXO1a acetylation [368], and decrease the transcriptional activity of FoxO1 [369].

Although phosphorylation and prevention of nuclear trafficking of FoxOs can potentially promote anti-aging pathways [331], FoxOs also have a beneficial side that can be linked to autophagy pathways [18,309]. Atherosclerosis can be lessened with FoxO1 and autophagy activity [259,370]. In experimental studies with Huntington’s disease (HD), increased activity of autophagy with FoxO1 can limit neuronal Huntington (mHtt) protein deposition [371]. Exercise-induced activation of autophagy results in the down-regulation of FoxO3a and suppression of sarcopenia [232]. Autophagy induction in association with modulation of FoxO signaling also results in decreased renal tubulointerstitial fibrosis [315] and protection against cardiotoxicity during ferroptosis [298].

In relation to MS, transcription factors, such as FoxO1, can impact brain myelination and support oligodendrocyte growth [372]. In the presence of enhanced activation of FoxO3a, inflammation in the brain tissue can ensue with cytokine release and apoptosis activation [373]. In older individuals, neuronal apoptosis and DNA destruction has been associated with nuclear transcription of target genes by FoxO3a [374]. As a result, therapeutic strategies would consider inhibition of FoxO DNA transcription through post-translational phosphorylation and exclusion from nuclear trafficking. However, autophagy activation may have protective effects during MS. Scenarios exist that can enhance neuronal and vascular survival through combined autophagy induction and activation of FoxOs. Autophagy with FoxO activity, such as during HD, can remove cellular deposits that would otherwise result in cell death [74,375]. In addition, the absence of FoxO3a may be detrimental and represent a lost checkpoint, since relapse in MS may occur with osteopontin and T cell activation under such conditions [376].

Other studies suggest that FoxOs in combination with silent mating type information regulation 2 homolog 1 (*Saccharomyces cerevisiae*) (SIRT1) may lead to immune dysregulation and neuronal inflammation during MS [377] (Figure 1). SIRT1 is a member of the sirtuin family (sirtuin 1) and is a histone deacetylase [2,5,98,106,195,299,308,378,379,380,381,382]. SIRT1 oversees DNA transcription by transferring acetyl groups from ε-*N*-acetyl lysine amino acids to the histones of DNA. FoxO proteins are deacetylated by SIRT1 as well as other histone deacetylases [18,299,313,321,383,384,385]. SIRT1 decreases oxidative stress, offers protection to neurons, and can preserve memory function [5,76,78,98,106,380,386,387]. SIRT1 maintains mitochondrial function in conjunction with other pathways in experimental models of neurodegeneration [388]. SIRT1 also prevents memory loss during oxidative stress in murine experimental models [389]. In part, SIRT1 activity can block FoxO to prevent cell injury [22,378]. However, SIRT1 is also controlled at times by FoxO proteins in feedback pathways. FoxOs bind to the SIRT1 promoter region to alter forkhead transcription. This promoter region contains a cluster of five putative core binding repeat motifs (IRS-1) and a forkhead-like consensus-binding site (FKHD-L). As an example, FoxO proteins are necessary for pre-implantation embryo development and control SIRT1 protein expression through autofeedback pathways [390]. FoxO proteins, such as FoxO1, also can modulate SIRT1 transcription and increase SIRT1 expression [391]. FoxOs and SIRT1 work synergistically to increase cell survival. SIRT1 and FoxO3a have been shown to limit Aβ injury that affects mitochondria and reduce oxidative stress toxicity [392].

## 7. The Mechanistic Target of Rapamycin and Multiple Sclerosis

MS and demyelinating disease can be significantly impacted by the mechanistic target of rapamycin (mTOR) pathways (Figure 1). mTOR is a 289-kDa serine/threonine protein kinase that is encoded by a single gene, *FRAP1* [13,46,71,73,98,124,243,244,393] (Table 1). mTOR is also known as the mammalian target of rapamycin and the FK506-binding protein 12-rapamycin complex-associated protein 1 [22,98,229]. Initially, mTOR was reported in *Saccharomyces cerevisiae* with *TOR1* and *TOR2* genes [124]. Both mTOR and TOR are inhibited by rapamycin, a macrolide antibiotic in *Streptomyces hygroscopicus* [229]. mTOR Complex 1 (mTORC1) and mTOR Complex 2 (mTORC2) use mTOR as a central component [71,96,100,308,394]. mTORC1 consists of the proline-rich Akt substrate 40 kDa (PRAS40), Deptor (DEP domain-containing mTOR interacting protein), mammalian lethal with Sec13 protein 8, termed mLST8 (mLST8), and Raptor [71,98]. mTOR oversees Raptor, which rapamycin can inhibit. Rapamycin can associate with immunophilin FK-506-binding protein 12 (FKBP12), which connects to the FKBP12 -rapamycin-binding domain (FRB) at the carboxy (C) -terminal of mTOR to block activity of the FRB domain of mTORC1 [22]. However, other possibilities for inhibition of mTORC1 activity exist that consist of Akt and p70 ribosomal S6 kinase (p70S6K) inhibitory phosphorylation, and catalytic domain allosteric alterations [395]. It is believed that mTORC2 disassembly is necessary with long-term administration of rapamycin to achieve activity inhibition equal to rapamycin inhibition of mTORC1. Deptor associates with both ataxia-telangiectasia (ATM), the transactivation/transformation domain-associated protein of mTOR, and the FAT domain (FKBP12 -rapamycin-associated protein) (FRAP) to block mTOR and mTORC1 activity. PRAS40 interferes with binding of p70S6K, mTORC1, and the eukaryotic initiation factor 4E (eIF4E)-binding protein 1 (4EBP1) with Raptor to block the activity of mTORC1 [22,98,219,396,397]. Acting as a checkpoint in this pathway, Akt influences mTORC1 activity to phosphorylate PRAS40, and inhibits it to increase mTORC1 activity [15,340,365]. PRAS40 is released from Raptor, and PRAS40 is subsequently maintained in the cytoplasm associated to the 14-3-3 protein [398,399,400]. mLST8 can enhance mTOR activation [22]. mLST8 facilitates Raptor in binding to 4EBP1 and p70S6K [401]. In contrast to mTORC1, mTORC2 consists of Rictor, Deptor, the mammalian stress-activated protein kinase interacting protein (mSIN1), mLST8, and the protein observed with Rictor-1 (Protor-1) [22,98,382,402,403]. mTORC2 controls changes in the cytoskeleton that involves protein kinase C-α (PKC-α) and migratory effects of cells with the Rac guanine nucleotide exchange factors P-Rex1 and P-Rex2, as well as Rho signaling. A protein kinase A/protein kinase G/protein kinase C (AGC) family member, glucocorticoid induced protein kinase 1 (SGK1) activity is fostered by mTORC2. Activity of SGK1 can also be enhanced by Protor-1 [404,405]. mTORC2 assembly and the subsequent phosphorylation of Akt is controlled by mSin1 [406]. Cell survival can be enhanced during mSIN1 and Rictor phosphorylation of Akt at serine^473^, and lead to threonine^308^ phosphorylation by phosphoinositide-dependent kinase 1 (PDK1).

mTOR can influence programmed cell death pathways through multiple mechanisms. mTOR activity can block apoptotic cell death in the nervous system [15,69,71,73,85,115,172,276,407], prevent oxidative stress injury [399,408,409,410], modulate the progression of infectious agents [85,411,412,413,414,415,416], and oversee metabolic homeostasis [72,115,417,418]. In the presence of mTOR activity, Aβ toxicity can be blocked [154,172,399,409,419,420,421], vascular cell death is prevented [105,422], and neonatal and adult central nervous system hypoxic injury is prevented [276,423]. Furthermore, neuronal differentiation is promoted [424], microglia survival is increased during oxidative stress exposure and Aβ toxicity [117,399,409,420,425,426], and neuroplasticity is fostered [48,310,427,428].

In regard to cognitive pathways, mTOR can oversee cellular metabolism to improve cognition. mTOR may provide protection through an improved nutritional balance and Mediterranean dietary regimen. mTOR can limit Aβ-induced astrocyte and non-neuronal cell injury through enhanced Akt activity during consumption of polyphenol of olives and olive oil that may be linked to the prevention of AD [429]. mTOR can modulate insulin physiology in neurodegenerative studies to increase survival of astrocytes [429], block hyperglycemic endothelial cell injury [430], and preserve metabolic regulation [431]. As a component of the mTOR pathway, the AMP-activated protein kinase (AMPK) modulates cellular metabolism [106,169,198,237,378,381,382,394,432,433], and the activation of AMPK reduces cognitive loss in studies of DM and AD [434,435], removes cerebral Aβ [436] and tau [437], limits Aβ neurotoxicity [392], diminishes long-term inflammation in in the nervous system [10,71,438,439], and fosters pathways for healthy aging [6,440,441].

Autophagy pathways are also critical for impacting neurodegeneration and usually involve the blockade of mTOR activity for neuroprotective pathways [17,22,44,70,100,105,169,242,243,244,259,293]. The induction of autophagy, which may require an mTOR blockade, can protect neuronal and non-neuronal cells [10,24,73,89,106,172,224,442]. For example, diseases of the retina may require mTOR inhibition with rapamycin to prevent retinal degeneration during MS DM [46]. Inflammatory pathways that involve peripheral blood mononuclear cells in MS also may require inhibition of mTOR pathways with rapamycin [443]. In experimental MS models, rapamycin with mTOR inhibition can prevent the clinical course of both relapsing-remitting and chronic experimental autoimmune encephalomyelitis, suggesting important clinical applications for the treatment of MS [201]. Rapamycin, through the blockade of cytokine release and the inhibition of a microglia immune response, has been shown to reduce clinical symptomatology and inflammatory responses in models of experimental autoimmune encephalomyelitis [134]; it has been suggested that inhibition of mTOR pathways may be necessary to reduce the risk of MS development [135]. Autophagy activation during decreased mTOR activity can maintain mitochondrial function [444], prevent injury to dopamine-dependent cells [445], provide neuroprotection with glutamine-dependent mechanisms [446], and decrease ROS release [447]. The improvement of memory function and cognition also may be linked to the maintenance of cellular glucose homeostasis. In the presence of limited mTOR activity, autophagy activation can lead to removal of Aβ, reduction of cognitive loss, and enhanced insulin-glucose metabolism [448]. Reductions in memory loss can be promoted through nutritional changes that focus on calorie reduction to reduce mTOR, increase autophagy activation [449], and foster microglial cell activity that can be altered by serum glucose changes [450]. Cognition may be improved with autophagy induction and reduced mTOR to remove tau [437], while loss of a necessary autophagy balance can contribute to dementia [156].

However, it is important to recognize that cognitive function as well as neuroprotection relies upon a careful balance between the activity of apoptosis and autophagy. mTOR and autophagy blockades are required for brain interneuron progenitor development [451]. Autophagy activation during high serum glucose levels can result in oxidative stress through mitochondrial dysfunction [94,380,441,452,453,454], lead to progenitor endothelial cell injury, and prevent new blood vessel growth [455]. Under some circumstances, autophagy can result in neuronal cell death [375,456,457]. Dysfunction or loss of mTOR signaling may at times result in cognitive impairment [13,18,73,105,117,458]. Furthermore, trophic factors such as EPO lead to enhanced neurovascular cell survival through activation of mTOR and reduction in autophagy [187,273,340]. EPO controls Akt and PRAS40 as well fosters neuronal cell health [137,398,459,460].

In regard to MS, mTOR can importantly impact inflammatory pathways that lead to neurodegeneration [3,73,85,211,244,285,461,462]. In clinical studies, mTOR pathway molecules may play an important role in determining the onset and progression of MS in patients [135]. Current therapies exist with metformin and biguanides that can impact neurodegenerative disease, and include demyelinating disease and cognitive loss [85,115,132,463]. Metformin blocks mTOR activity to foster autophagy. However, it is known that metformin can also act independent of AMPK pathways [464]. Inhibition of mTOR activity with AMPK activation during metformin treatment can support myelin growth through the reduction of oxidative stress in oligodendrocytes [132]. These observations with metformin also appear to promote activity of oligodendrocytes that can lead to myelination and repair in the nervous system [132]. In regard to risk factors for MS, metformin can limit impaired function in overweight individuals or those suffering from DM when exposed to COVID-19 [465,466]. In models of autoimmune encephalomyelitis, the modulation of mTOR and autophagy activity can affect activated microglia, reduce the release of cytokines, and potentially modulate inflammation and demyelination in the nervous system [134]. It is important to note that since the loss of SIRT1 activity may be involved in immune dysregulation during MS [377], mTOR has a complex relationship with SIRT1 [22]. SIRT1 can require limited mTOR activity to support neuronal development in the presence of low nutritional circumstances [467]. During ROS release and oxidative stress, a combination of SIRT1 activation, autophagy induction, and reduced mTOR is necessary for the function of embryonic stem cells and organelles such as mitochondria [468]. Inhibition of mTOR with SIRT1 activation can increase photoreceptor cell survival [469] and limit cell senescence [470]. However, at certain times, SIRT1, mTOR, and FoxOs may be necessary for cell survival, since protection of neurons in the dopamine system requires complementary activities for SIRT1, FoxOs, and mTOR [471].

## 8. Conclusions and Future Considerations

Lifespan expectancy is rising throughout the world. As a result, the prevalence of neurodegenerative disorders that affect more than fifteen percent of the global population and comprise greater than six hundred disease entities is increasing. With the increased age of the population and underlying cellular mechanisms such as cell senescence and TL impairment, dementia has now become the 7th leading cause of death in the world. Given this knowledge, increased focus is now directed to individuals with MS, a disorder that affects a significant proportion of the world’s population. Individuals with MS are now recognized as being significantly impacted by cognitive loss and dementia. It is believed that loss of cognitive function in MS occurs in approximately sixty-five percent of individuals and can affect processing of information, attention, and memory recall. Multiple cellular mechanisms may lead to onset and progression of MS, such as inflammatory mediators, demyelination and remyelination pathways, oxidative stress, blood-brain barrier impairment, viral antigens, and cellular metabolism dependent upon nicotinamide adenine dinucleotide (NAD+). At present, DMTs can only limit the rate of relapse in MS patients, but cannot prevent disease progression. Of further concern, early initiation of DMTs may slow the progression of brain volume loss, but cognitive disability may continue to progress. New and innovative avenues for the investigation and treatment of demyelinating disorders are required that involve autophagy, apoptosis, FoxOs, mTOR, AMPK, SIRT1, and related systems with the APOE-ε4 gene and SARS-CoV-2.

For the pursuance of new strategies that can address cognitive loss in MS, APOE and infection with SARS-CoV-2 may be significant risk factors for MS. Interestingly, risk factors for cognitive loss in MS share similarities with other cognitive disorders such as AD. In patients with optic neuritis, APOE serum levels are markedly higher than in control patient groups, and the *APOE* ε3/ε3 genotype may increase the risk of developing optic neuritis in males. Other risk factors such as SARS-CoV-2 may be a risk factor for developing cognitive loss in MS. Memory loss can develop after infection with SARS-CoV-2 [85,115,283,472,473,474]. APOE-ε4 is associated with long-COVID disability and loss in cognition [110,155,475]. Two ε4 alleles of APOE-ε4 confers a loss in gene activity that can defend against viral infections leading to disruption of cerebral blood vessels and increased inflammatory activity [155,192]. As a result, individuals with APOE may experience demyelination, cognitive loss, and increased death rates during a SARS-CoV-2 infection [10,15,22,33,38,51,258,277,433,476,477].

Pathways with programmed cell death in MS have intricate relationships with FoxOs, mTOR, and SIRT1. Autophagy, apoptosis, pyroptosis, and ferroptosis may have an important role in the regulation of oligodendrocyte development, myelination, inflammasome expression, intracellular iron accumulation, activation of microglia, and cell survival during oxidative stress. However, these relationships are complex and may require a fine balance. For example, although enhanced FoxO3 activity alone may foster disease progression in MS by resulting in inflammation, cytokine activation, and neuronal cell apoptosis, FoxO activation in combination with autophagy during these circumstances may be protective during MS. FoxO3a loss also may be detrimental and represent a lost checkpoint, since MS recurrence may ensue with osteopontin and T cell activation under these conditions. These studies suggest that activation of FoxO with complementary autophagy induction may be necessary for protective pathways in MS. FoxO proteins are also dependent upon SIRT1, and function through autofeedback mechanisms to regulate SIRT1 activity. FoxOs and SIRT1 can work synergistically to increase cell survival and reduce oxidative stress toxicity. In a similar vein, mTOR inhibition with autophagy activation can provide neuroprotection, decrease ROS release, increase astrocyte viability, and preserve glucose homeostasis. However, mTOR and autophagy blockades are required for brain interneuron progenitor development, and the dysfunction or loss of mTOR signaling can lead to cognitive impairment. Furthermore, SIRT1, mTOR, AMPK, and FoxOs may be necessary for cell survival that involves agents such as nicotinamide and EPO, as well as for dopaminergic neuronal cell survival that requires complementary activities for SIRT1, FoxOs, and mTOR. The pathways of programmed cell death, FoxOs, mTOR, AMPK, and SIRT1 offer great promise for the understanding and treatment of cognitive loss in MS, but future investigations will be necessary to further understand the complexity of these pathways to achieve long-lasting beneficial outcomes.

## Figures and Tables

**Table 1:** Highlights.

Cognitive Impairment in Multiple Sclerosis
Multiple sclerosis (MS) is considered to be the most common demyelinating disorder that affects the immune system, and almost three million individuals suffer from MS throughout the world. Loss of cognitive function in MS occurs in at least sixty-five percent of individuals and can affect processing of information, attention, memory recall and other executive functions. Novel strategies for the treatment of cognitive loss in MS involve programmed cell death, mammalian forkhead transcription factors (FoxOs), the mechanistic target of rapamycin (mTOR), AMP activated protein kinase (AMPK), the silent mating type information regulation 2 homolog 1 (*Saccharomyces cerevisiae*) (SIRT1), and associated pathways with the apolipoprotein E (APOE-ε4) gene and severe acute respiratory syndrome coronavirus (SARS-CoV-2). Risk factors for cognitive loss and disability in MS consist of exposure to infectious agents, such as SARS-CoV-2 as part of coronavirus disease 2019 (COVID-19), that may lead to increased death rates in patients with MS. APOE-ε4 has been associated with difficulties with cognitive function and an increased risk of developing optic neuritis. Autophagy, apoptosis, pyroptosis, and ferroptosis play a significant role in MS during the generation of reactive oxygen species (ROS) and oxidative stress, mitochondrial dysfunction, intracellular iron accumulation, activation of microglia, demyelination, and neuronal axonal loss that ultimately can impair cognitive function. Although these pathways may function in opposition at times, FoxOs, mTOR, AMPK, and SIRT1 have complex interactions that involve autofeedback pathways and may function at times synergistically to maintain cell survival function that can involve agents such as nicotinamide and EPO.

## Data Availability

Not applicable.
